# Prof. Rostan on Cerebral Affections

**Published:** 1847-12

**Authors:** 


*Prof. Rostan on Cerebral Affections.*—M. Rostan is in the habit, every year, of devoting a few clinical lectures to the special illustration of diseases of the brain, in which department of science, as you are aware, he has done more than any living physician. In one of his late lectures he examined the value of paralysis as a diagnostic sign. His remarks may be summed up as follows:

Paralysis is one of the most striking and unerring signs which can exist to mark the seat of cerebral lesions. It should be studied in its seat, extent, intensity, march, duration, and in all the different circumstances which may impart to it different characters.—The seat of the paralysis indicates, in the first place, the part of the brain where we find the organic alteration; but much is required to be able, after that, to determine precisely in every case the extent of this alteration. If a circumscribed paralysis corresponds to a circumscribed cerebral alteration, it is possible that this paralysis, occupying both the arm and leg of the same side, may be produced by a simple lesion of small extent, situated upon the confines of those portions of the brain which preside over the movements of the two members, and act equally upon the one and the other. In the appreciation of the extent of the lesion, you must take into account also the extent of the paralysis; but you must not think that in this respect the relations are mathematical; for an organic lesion of circumscribed extent may produce a very complete paralysis, as, on the other hand, an extensive lesion may give rise to a paralysis so slight, that one may even in some degree doubt whether there really exists any anatomical disorder or change. When I first discovered *ramollissement* of the brain, I feared that it would not be possible, during life, to distinguish this from other alterations of that organ—that, occupying the same situation, it would manifest itself by the same phenomena.

But the mode of invasion, the march of the disease, will not be the same as those, for example, of cerebral hemorrhage. They differ in effect; whence the necessity of bearing in mind the march of the affection. If the paralysis is sudden and without precursory phenomena, it is extravasation of blood—cerebral hemorrhage—that you should suspect in the great majority of cases, remembering at all times that there are exceptions to the rule. Thus in certain cases of *ramollissement*, while the lesion is not very extensive, it may produce no sensible trouble; but the day comes when its volume is such that suddenly the phenomena appear with fearful rapidity. This is the drop of water that overruns the vase. The analogy is true. As there are *ramollissements* without precursory phenomena, so these phenomena may occasionally precede the congestion or cerebral hemorrhage.

After examination of the seat and the extent and precursory phenomena, you must look at the march of the affection. A paralysis declares itself, and augments day by day; suddenly there may supervene a diminution of the symptoms, a passing amelioration, and this is a circumstance which may lead you into error; for paralysis, even when increasing, has not always a regular march. Under the influence of a sanguine emission, the compression of the pulp may momentarily cease, and consequently the paralysis diminish. This now explains how there may be greater or less alteration in the intensity of paralysis. The paralysis which succeeds cerebral hemorrhage may disappear or persist, according to the manner in which the effusion is made. When the blood has simply separated the cerebral fibres, you can easily understand that in the proportion and measure of its absorption the phenomena of paralysis will disappear; when it has destroyed the cerebral fibres, then the power of motion is wholly lost, and it is in vain that you employ electricity, nux vomica, blisters, etc.

I remember two curious cases of paralysis analogous to some of which you have perhaps read. One was a subject who had been paralytic for many years; he could not walk; his house took fire; he sprang from his bed, walked alone to escape being burned, and was cured. A hemiplegic woman was received at Salpetriere.—Some of her acquaintances put her in communication with a miracle-worker, Prince Hoenlohe. Prayers were said for nine days; at the end of the time predicted by the prince, the patient was cured, having recovered her movements. This case made so much noise that the governors of the hospitals believed it their duty to institute an inquiry, and they asked me my opinion. My reply was simple, and destroyed all the marvelousness of the miracle. In the first place, it is possible that a moral cause might act upon the brain like the blow of a club upon the shoulder, and that there may not have been a material destruction of the encephalic substance. In other cases there have been hemorrhages, effusion between the cerebral fibres; the effusion is absorbed, and motion does not return, simply because the patient has lost the habit of exercising his limb. Let a cause, it makes no difference what, compel this movement, and the patient is cured. With this woman two things were possible: either that she was in this last condition, and the persuasion that she would be cured had actually given her this movement; or, what is also supposable, she had simulated paralysis in order to enter the hospital, and not finding it quite as agreeable a place as she supposed it would be, became weary of her situation and resolved to change it.

From the nature of the lesion by which it has been produced, it follows that paralysis terminates by recovery, or increases, or remains stationary, and that the treatment should be directed not against the paralysis itself, which is but a sympton, but against the lesion which has given rise to it.—*Notes on Medical matters in Paris, in Western Journal of Medicine and Surgery.*

